# Genetic polymorphism in European and African sheep breeds reared in Hungary based on 48 SNPs associated with resistance to gastrointestinal parasite infection using KASP-PCR technique

**DOI:** 10.1007/s11250-023-03609-0

**Published:** 2023-05-09

**Authors:** Putri Kusuma Astuti, Dinu Gavojdian, Daniela Elena Ilie, George Wanjala, István Monori, Zoltán Bagi, Szilvia Kusza

**Affiliations:** 1 Centre for Agricultural Genomics and Biotechnology, University of Debrecen, Debrecen, 4032 Hungary; 2 Doctoral School of Animal Science, University of Debrecen, Debrecen, 4032 Hungary; 3 Department of Animal Breeding and Reproduction, Faculty of Animal Science, Universitas Gadjah Mada, Yogyakarta, 55281 Indonesia; 4 Research and Development Institute for Bovine Balotesti, 77015 Balotesti, Ilfov Romania; 5 Research and Development Station for Bovine, 310059 Arad, Romania; 6 Alpha seed Ltd., Karcag, 5300 Hungary

**Keywords:** KASP PCR, *Ovis aries*, Parasite resistance genes, Polymorphism, TLRs

## Abstract

This pilot study used an alternative and economically efficient technique, the Kompetitive Allele-Specific Polymerase Chain Reaction (KASP-PCR) to examine 48 SNPs from 11 parasite-resistance genes found on 8 chromosomes in 110 animals from five sheep breeds reared in Hungary; Hungarian Tsigai, White Dorper, Dorper, Ile de France, and Hungarian Merino. Allele and genotype frequencies, fixation index, observed heterozygosity, expected heterozygosity, *F* statistic, and their relationship with the Hardy–Weinberg equilibrium (WHE) and the polymorphic information content (PIC) were determined, followed by principal component analysis (PCA). As much as 32 SNPs out of the 48 initially studied were successfully genotyped. A total of 9 SNPs, 4 SNPs in *TLR5*, 1 SNP in *TLR8*, and 4 SNPs in *TLR2* genes, were polymorphic. The variable genotype and allele frequency of the TLRs gene indicated genetic variability among the studied sheep breeds, with the Hungarian Merino exhibiting the most polymorphisms, while Dorper was the population with the most SNPs departing from the HWE. According to the PIC value, the rs430457884-*TLR2*, rs55631273-*TLR2*, and rs416833129-*TLR5* were found to be informative in detecting polymorphisms among individuals within the populations, whereas the rs429546187-*TLR5* and rs424975389-*TLR5* were found to have a significant influence in clustering the population studied. This study reported a moderate level of genetic variability and that a low to moderate within-breed diversity was maintained in the studied populations.

## Introduction

Small ruminants account for a substantial percentage of the worldwide livestock sector, with estimates of 1173 million heads for sheep and 1003 million heads for goats, respectively (FAO 2018). Sheep in Hungary is the third largest livestock population species, after poultry and pigs (Hungarian Central Statistical Office 2020). Hungary’s vast pastureland and temperate climate are excellent for sheep farming, making this country a significant sheep producer in the past, during nineteenth-century Europe. However, the changing political situations and agricultural activities in 1989 had a massive impact on Hungarian sheep farming, and the lowest point in sheep farming was recorded in 1996 (Nagy et al. 2011; Beke-Lisányi 2018). According to EUROSTAT (2022), Hungary’s sheep population was 1,061 thousand heads in 2019, with more than 90% of the breed structure being represented by Merino or Merino-derived breeds (Fésüs et al. 2008), with the remainder filled by several other imported European and African breeds such as Dorper, White Dorper, and Ile de France, as well the indigenous Hungarian Tsigai and Racka are also commonly reared in Hungary (Kukovics and Jávor 1999). In recent years, numerous efforts have been undertaken to revitalize sheep production using several approaches, one of which is through increasing production levels through better animal health and pasture management.

One of the main causes of production losses in small ruminants worldwide is represented by parasitic infections in grazing animals. For instance, Charlier et al. (2020) estimated that helminth infections cost € 151 million/year in dairy sheep and € 206 million/year in meat sheep farming in the European countries. Furthermore, Mavrot (2016) previously estimated higher costs of € 157–477 million/year in sheep meat farming, based on individual European country reports. These findings evidenced a significant economic loss caused by parasite infection in sheep.

Gastrointestinal parasitism’s impact on the health and production levels of grazing ruminants are well documented and includes significant weight and body condition losses and decreased milk yields (Carta et al. 2009; van der Voort et al. 2014; Ertaş et al. 2022), diarrhea (Jacobson et al. 2020), anorexia (al Kalaldeh et al. 2019), anemia (Mongruel et al. 2020), and higher levels of mortality Underwood et al. 2015). Furthermore, with the threat of climate change, the prevalence of parasite infection in sheep is expected to increase, even in regions where such aspects were not significantly problematic up to date. For instance, the seasonality of helminth parasites is expected to change as a result of warmer temperatures, and while the parasite survival rates during wintertime in temperate climates are expected to improve (Short et al. 2017). Moreover, increased rainfall levels are predicted to increase the prevalence of the liver fluke, *Fasciola hepatica* (Shrestha et al. 2020), *Leishmania* (Short et al. 2017), and several tick-borne *Hemiparasitic* illnesses such as *Anaplasma* spp., *Babesia* spp., and *Theileria* spp., which have been demonstrated to be influenced by changes in temperature and humidity (Abdullah et al. 2019).

Limited studies have been made focusing on gastrointestinal parasite infections in sheep reared in Hungary. Tóth et al. (2019) reported the most prevalent parasitic nematodes burden in Hungary to consist mainly out of *Protostrongylus* sp. and *Strongylus* sp., tapeworm *Moniezia* sp. and the *Coccidian eimeria* sp. Understanding genetic diversity and susceptibility for gastrointestinal parasitism resistance would be an excellent starting point, given that the variability in genetics within a population is fundamental to the immune response during infectious diseases. Up-to-date, emphasis has been made on the significance and potential benefits of heterogeneous genetic populations, notably in terms of the complex reactions they confer to epidemics, their longevity, and general resilience (Springbett et al. 2003; Kristensen et al. 2015). The preservation of genetic heterogeneity in livestock populations is critical to maintaining healthy livestock practices and for the preservation of biodiversity, considering the genetic advantages that locally adapted breeds have in reacting to epidemiological outbreaks.

The current pilot study in gastrointestinal parasite infection related genes in African and European sheep breeds reared in Hungary investigated the polymorphism of 48 SNPs belonging to 11 genes related to genetic gastrointestinal parasite resistance in five European and African sheep breeds which currently are concerned as part of Hungary’s sheep genetic selection and improvement program: Hungarian Tsigai, White Dorper, Dorper, Ile de France, and Hungarian Merino. The Hungarian Merino and the Hungarian Tsigai are two indigenous breeds of sheep vital to the Hungary’s efforts to preserve its genetic resources in the face of rising global demand for sheep genetics due to climate change. The Hungarian Merino sheep breed accounts for more than 80% of Hungary’s total sheep population. There were less than 5000 Hungarian Merinos left in 2014, rendering them an endangered species. Although not as numerous as Hungarian Merino, Hungarian Tsigai has remained a consistent component of the local livestock (< 10%) over the last two centuries.

In this study, the Kompetitive Allele Specific Polymerase Chain Reaction (KASP-PCR) was used. Fluorescence resonance energy transfer (FRET) generates signals in KASP-PCR. Two luminous cassettes detect bi-allelic SNP allele-specific amplification (Suo et al. 2020). Biallelic characterization of SNPs, insertions, and deletions in specific loci is easy, fast, and inexpensive (Alvarez-Fernandez et al. 2021). In the first round of PCR, allele-specific primers match the target SNP, and the common reverse primer amplifies the target area. The fluor-labeled oligos keep connected to their quencher-labeled complementary oligos, preventing fluorescence. The allele-specific primer is integrated into the template. As the PCR progresses, a fluor-labeled oligo corresponding to the amplified allele is incorporated into the template and no longer linked to its quencher-labeled complement, forming an adequate fluorescent signal (He et al. 2014).

Understanding the genetic diversity of sheep breeds will aid in understanding the significance of genetic variants in parasite resistance. In addition, the findings of the current study might be used for future sheep genetic improvement programs in Europe and Africa, as well as for future conservation initiatives, in order to improve sheep productivity, health, and animal welfare.

## Materials and methods

### Genomic DNA extraction

Blood samples were collected from 110 indigenous and non-indigenous sheep, as follows: Hungarian Tsigai = 10 and Hungarian Merino = 50 as the indigenous breed, and White Dorper = 10, Dorper = 10, and Ile de France = 30 as the non-indigenous breeds. Although the sample population was relatively low because this is a pilot project, all of the animals selected were genetically unrelated, and sampling was conducted in several different farms to better represent the breed. The method of Zsolnai and Orbán (1999) for isolating genomic DNA from blood was utilized. The DNA was stored at − 20 °C until it was analyzed. A NanoDrop Spectrophotometer was used to assess the concentration and quality of DNA (Thermo Scientific, Waltham, MA, USA). All samples were diluted to a uniform concentration before genotyping, which was done with 50 ng of DNA per sample.

### Selection of SNPs

A panel of 48 SNPs from 11 parasite-resistance genes located on 8 chromosome was chosen based on previous genome-wide association studies (GWAS) and marker-assisted selection studies (Carracelas et al. 2022; Oget et al. 2019; Archibald et al. 2010) across the sheep genome (Table 1). The SNP data for Ovis were obtained from the NCBI’s Single Nucleotide Polymorphism Database (dbSNP http://www.ncbi.nlm.nih.gov) or Ensembl (http://www.ensembl.org). In the end, only 32 SNPs out of the 48 initially studied were successfully genotyped.

**Table 1:** Details of SNP ID, gene, and chromosome at the 48 SNPs panel used in the study

Code	SNP ID	Locus	Chromosome	Allele
SNP1	rs429546187	*TLR5*	12	*C/G*
SNP2	rs403288183	*TLR8*	X	*C/T*
SNP3	rs401390846	*TLR2*	17	*C/A*
SNP4	rs424975389	*TLR5*	12	*A/C*
SNP5	rs418959585	*TLR5*	12	*G/A*
SNP6	rs160387232	*IL1R1_2*	3	*C/T*
SNP7	rs590620426	*IL2_1*	17	*C/G*
SNP8	rs596312311	*IL2_2*	17	*C/T*
SNP9	rs419524929	*CRYL1*	10	*C/T*
SNP10	rs160515511	*CRYL1*	10	*G/C*
SNP11	rs160515510	*CRYL1*	10	*G/A*
SNP12	rs160515503	*CRYL1*	10	*A/G*
SNP13	rs160515483	*CRYL1*	10	*G/A*
SNP14	rs594194839	*CRYL1*	10	*A/G*
SNP15	rs160515477	*CRYL1*	10	*A/G*
SNP16	rs55632294	*TLR2*	17	*T/C*
SNP17	rs55632297	*TLR2*	17	*A/G*
SNP18	rs55631273	*TLR2*	17	*C/T*
SNP19	rs430457884	*TLR2*	17	*A/G*
SNP20	rs160821602	*TLR2*	17	*T/G*
SNP21	rs162073322	*TLR2*	17	*A/G*
SNP22	rs162136344	*RARα*	11	*C/G*
SNP23	rs160601236	*MAPRE1*	13	*A/G*
SNP24	rs160601254	*MAPRE1*	13	*A/G*
SNP25	rs420375907	*TLR5*	12	*A/G*
SNP26	rs399647577	*TLR5*	12	*C/T*
SNP27	rs412232316	*TLR5*	12	*A/C*
SNP28	rs416833129	*TLR5*	12	*A/G*
SNP29	rs593219494	*TLR8*	X	*A/C*
SNP30	rs159830151	*SOCS2*	3	*G/A*
SNP31	rs868996547	*SOCS2*	3	*T/C*
SNP32	rs159830157	*SOCS2*	3	*G/A*
SNP33	rs160515509	*CRYL1*	10	*C/T*
SNP34	rs161992521	*CRYL1*	10	*C/A*
SNP35	rs413718437	*CRYL1*	10	*T/C*
SNP36	rs55631283	*TLR2*	17	*A/G*
SNP37	rs398141211	*TLR2*	17	*G/A*
SNP38	rs597393983	*SLC40A1*	2	*G/T*
SNP39	rs159515567	*SLC40A1*	2	*G/A*
SNP40	rs405524806	*SLC40A1*	2	*C/T*
SNP41	rs399765789	*MAPRE1*	13	*T/G*
SNP42	rs405485855	*TLR5*	12	*G/T*
SNP43	rs410533606	*TLR5*	12	*G/A*
SNP44	GQ175930	*TLR7*	X	*C/A*
SNP45	GQ175929	*TLR7*	X	*G/A*
SNP46	GQ175927	*TLR7*	X	*A/G*
SNP47	GQ175932	*TLR7*	X	*G/A*
SNP48	rs161696362	*TLR8*	X	*C/G*

### Genotyping and quality control

KASP PCR (KASPTM, LGC Genomics, Teddington, Middlesex, UK) was used to perform bi-allelic discrimination of the selected 48 SNPs. The data were visualized using SNP Viewer software version 1.99 (Hoddesdon, UK). All genotype data were exported for statistical analysis. Only SNPs that were found in at least 90% of the breeds were included. Quality control of genotyped data comprised of eliminating a number of animals with more than 10% missing SNP calls and a number of SNPs with call rates less than 90%, resulting in disparities for the number of individuals among SNPs.

### Data analysis

The raw allele calls provided from LGC Genomics were examined using LGC Genomics’ KlusterCaller program. POPGENE software version 1.31 (Yeh et al. 1999) was used to calculate allele and genotype frequencies, fixation index (Fis), observed heterozygosity (Ho), expected heterozygosity (He), *F* statistic (Fst), and their accordance with or deviation from the Hardy–Weinberg equilibrium(HWE). Polymorphic information content (PIC) was determined online at https://gene-calc.pl/pic.

To visualize the genetic divergences between sheep breeds, the principal component analysis (PCA) was performed using FactoMineR (Lê et al. 2008) and ggplot2 (Wickham 2016) tools from the R Program (R Core Team 2020).

## Results

### Genetic diversity

This work studied 48 SNPs in 11 genes related to gastrointestinal parasite resistance in five European and African sheep breeds using the KASP genotyping technique (Table 1). A number of 32 SNPs out of the 48 initially studied were successfully genotyped (66.67%), while 16 others failed. As many as 9 markers (18.75%) of the successfully genotyped were found to be polymorphic (Table 2). The monomorphic markers were removed from further investigation.

**Table 2:** The 9 polymorphic loci details

Code	SNP ID	Locus	Chromosome position	Chromosome	Allele	Sequence
SNP1	rs429546187	TLR5	24,624,977	12	C/G	AGCAGGAAGACTGTCTGTCTCGTGA[G/C]CAGACACTTCCTTAGAGACGGGTGG
SNP2	rs403288183	TLR8	10,404,549	X	C/T	CTACGAACTGAAAAAATATCCTCAG[T/C]ACATTAACATTTCCAAAAATTTCTC
SNP3	rs401390846	TLR2	3,844,163	17	C/A	CTTATAGATATTGTAAGTTCCTTAG[A/C]TTATTTAGAACTGAGAGATACTAAT
SNP4	rs424975389	TLR5	24,625,779	12	A/C	TCATCTCCAACTCCTCTATCTGAAT[C/A]AAAACTACCTGAATTTCCTTCCACC
SNP18	rs55631273	TLR2	3,842,829	17	C/T	GGAGCTGGAGCACTTCAACCCTCCC[T/C]TTAAGCTGTGTCTTCATAAGCGAGA
SNP19	rs430457884	TLR2	3,842,888	17	A/G	GACGCCTTTGTGTCCTACAGCGAGC[G/A]GGATTCCTACTGGGTGGAGAACCTC
SNP20	rs160821602	TLR2	3,843,675	17	T/G	CTCAGCCTGTGAGCATGCCTGGCCC[G/T]TCCTTCAAACCCTGGTTTTAAGGCA
SNP27	rs412232316	TLR5	24,625,722	12	A/C	TCATCTCCAACTCCTCTATCTGAAT[C/A]AAAACTACCTGAATTTCCTTCCACC
SNP28	rs416833129	TLR5	24,626,978	12	A/G	TTGACCATTTACAGAGAAGCCTTCC[A/G]AAACCTGCCCAATCTCAGGATCCTG

The identified allele calls from the nine polymorphic markers with a 99.80% allele call rate. Polymorphic markers were rs429546187-*TLR5*, rs403288183-*TLR8*, rs401390846-*TLR2*, rs424975389-*TLR5*, rs55631273-*TLR2*, rs430457884-*TLR2*, rs160821602-*TLR2*, rs412232316-*TLR5*, and rs416833129-*TLR5*. The polymorphic markers were discovered in *TLR5*, *TLR8*, and *TLR2* genes on chromosomes 12, 27, and 17, respectively. Varies genotype and allele frequency were observed (Table 3).

**Table 3:** Allele frequency, genotype frequency and Hardy Weinberg Equilibrium analysis for 9 polymorphic loci

SNP ID	Locus	Genotype	Allele	Hungarian Tsigai			White Dorper			Dorper			Ile de France			Hungarian Merino			Overall		
				Genotype Freq	Allele Freq	*x*^2^	Genotype Freq	Allele Freq	*x*^2^	Genotype Freq	Allele Freq	*x*^2^	Genotype Freq	Allele Freq	*x*^2^	Genotype Freq	Allele Freq	*x*^2^	Genotype Freq	Allele Freq	*x*^2^
rs429546187	*TLR5*	AA	A	0.000	0.000	ND	0.000	0.000	ND	0.000	0.200	0.502	0.000	0.000	ND	0.000	0.020	0.919	0.000	0.027	0.788
		GA	G	0.000	1.000		0.000	1.000		0.400	0.800		0.000	1.000		0.040	0.980		0.054	0.973	
		GG		1.000			1.000			0.600			1.000			0.960			0.945		
rs403288183	*TLR8*	CC	C	0.000	0.000	ND	0.000	0.150	0.656	0.100	0.450	0.252	0.200	0.433	0.715	0.000	0.080	0.567	0.063	0.209	0.188
		TC	T	0.000	1.000		0.300	0.850		0.700	0.550		0.467	0.567		0.160	0.920		0.291	0.791	
		TT		1.000			0.700			0.200			0.333			0.840			0.646		
rs401390846	*TLR2*	AA	A	0.300	0.550	0.893	0.400	0.650	0.882	1.000	1.000	ND	0.233	0.533	0.302	0.580	0.790	0.068	0.482	0.705	0.492
		CA	C	0.500	0.450		0.500	0.350		0.000	0.000		0.600	0.467		0.420	0.210		0.445	0.295	
		CC		0.200			0.100			0.000			1.167			0.000			0.073		
rs424975389	*TLR5*	AA	A	1.000	1.000	ND	1.000	1.000	ND	0.600	0.800	0.502	1.000	1.000	ND	0.960	0.980	0.919	0.945	0.973	0.789
		GA	G	0.000	0.000		0.000	0.000		0.400	0.200		0.000	0.000		0.040	0.020		0.055	0.027	
		GG		0.000			0.000			0.000			0.000			0.000			0.000		
rs55631273	*TLR2*	CC	C	0.200	0.450	0.893	0.600	0.800	0.502	0.400	0.500	0.034*	0.167	0.467	0.319	0.000	0.210	0.020*	0.155	0.382	0.662
		TC	T	0.500	0.550		0.400	0.200		0.200	0.500		0.600	0.533		0.420	0.790		0.455	0.618	
		TT		0.300			0.000			0.400			0.233			0.580			0.390		
rs430457884	*TLR2*	AA	A	0.000	0.150	0.656	0.000	0.450	0.015*	0.400	0.500	0.039*	0.000	0.000	ND	0.000	0.020	0.919	0.036	0.110	0.007*
		GA	G	0.300	0.850		0.900	0.550		0.200	0.500		0.000	1.000		0.040	0.980		0.145	0.890	
		GG		0.700			0.100			0.400			1.000			0.960			0.809		
rs160821602	*TLR2*	GG	G	0.700	0.850	0.656	1.000	1.000	ND	1.000	1.000	ND	1.000	1.000	ND	0.960	0.980	0.886	0.954	0.977	0.828
		TG	T	0.300	0.150		0.000	0.000		0.000	0.000		0.000	0.000		0.040	0.200		0.045	0.023	
		TT		0.000			0.000			0.000			0.000			0.000			0.000		
rs412232316	*TLR5*	AA	A	0.300	0.400	0.298	0.000	0.050	1.000	0.400	0.700	0.223	0.000	0.000	ND	0.000	0.200	0.919	0.064	0.114	0.000*
		CA	C	0.200	0.600		0.100	0.950		0.600	0.300		0.000	1.000		0.040	0.980		0.100	0.886	
		CC		0.500			0.900			0.000			1.000			0.960			0.836		
rs416833129	*TLR5*	AA	A	0.300	0.400	0.043*	0.000	0.050	1.000	0.200	0.500	0.641	0.000	0.000	ND	0.000	0.000	ND	0.045	0.087	0.000*
		GA	G	0.200	0.600		0.100	0.950		0.400	0.500		0.000	1.000		0.000	1.000		0.082	0.913	
		GG		0.500			0.900			0.200			1.000			1.000			0.863		

*x*^2^ Hardy–Weinberg equilibrium test; *ND* monomorphic; *p < 0.05

The genotype of homozygous *GG* in rs429546187-*TLR5* was found dominantly in all five populations studied, with the range of *G* allele frequency varying between 0.80 to 1.00, followed by the heterozygote *GA*, which was only found in Dorper and Hungarian Merino breeds. The same pattern was observed with the homozygote *AA* for rs424975389-*TLR5*, with *A* allele frequency of 0.80 to 1.00. For rs416833129-*TLR5*, the *G* allele was more frequent in all five populations, with an allele frequency ranging between 0.50 and 1.00, except for the Dorper, where the heterozygote *GA* was the most frequent. While for rs412232316-*TLR5*, the *C* allele was the most dominant in all populations, except for the Dorper breed, for which the genotypes *CA* and *AA* were more frequent, with an *A* allele frequency of 0.70.

For rs403288183-*TLR8*, the homozygote *TT* was found in most Hungarian Tsigai, White Dorper, and Merino individuals, with the allele frequency ranging between 0.92 and 1.00, while in Dorper and Ile de France breeds, the heterozygote *TC* was the most prevalent, with the homozygote *CC* being observed as well.

For the *TLR2* locus, there were four polymorphic markers observed. For the rs401390846, both alleles *A* and *C* were moderately found in Hungarian Tsigai (*A* = 0.550 and *C* = 0.450), White Dorper (*A* = 0.650 and* C* = 0.350), and Ile de France (*A* = 0.533 and *C* = 0.467), even though the A allele was dominant in Dorper and Merino breeds, with a frequency of 1.00 and 0.79, respectively. For the rs55631273, both alleles *C* and *T* were found moderately in each of the sampled populations, except for the White Dorper and Merino. In rs430457884, the genotype *GG* was highly observed in all populations, although the heterozygote *GA* was dominantly observed in the White Dorper population, while the *G* allele dominant for rs160821602 with the allele frequency varied between 0.85 and 1.00.

The Ho, He, PIC, and Fst values for the various breeds studied were determined and are listed in Table 4. The most polymorphic markers were found in rs55631273-*TLR2* (0.269–0.375), rs430457884-*TLR2* (0–0.375), and rs416833129-*TLR5* (0–0.375). In general, the obtained results showed that the PIC values were smaller than 0.5, with the minimum and maximum values of 0.044 and 0.361, suggesting that all investigated markers are considered moderately informative.

**Table 4:** Polymorphic information content (PIC), expected heterozygosity (He), observed heterozygosity (He), fixation index (Fis), *F* statistic (Fst), and sample size (*n*)

SNP	Locus		Hungarian Tsigai	White Dorper	Dorper	Ile de France	Hungarian Merino	Overall
rs429546187	*TLR5*	PIC	0.000	0.000	0.000	0.269	0.000	0.053
		H obs	0.000	0.000	0.400	0.000	0.040	0.545
		H exp	0.000	0.000	0.337	0.000	0.040	0.053
		n	20.000	20.000	20.000	60.000	100.000	220.000
		Fis	0.000	0.000	-0.250	0.000	-0.020	-0.025
		Fst						0.146
rs403288183	*TLR8*	PIC	0.000	0.223	0.373	0.371	0.136	0.276
		H obs	0.000	0.300	0.700	0.467	0.160	0.291
		H exp	0.000	0.268	0.521	0.499	0.149	0.332
		n	20.000	20.000	20.000	60.000	100.000	220.000
		Fis	0.000	-0.177	-0.414	0.050	-0.087	-0.172
		Fst						0.198
rs401390846	*TLR2*	PIC	0.373	0.352	0.000	0.374	0.277	0.329
		H obs	0.500	0.500	0.000	0.600	0.420	0.446
		H exp	0.521	0.479	0.000	0.506	0.335	0.418
		n	20.000	20.000	20.000	60.000	100.000	220.000
		Fis	-0.010	-0.099	0.000	-0.205	-0.266	-0.135
		Fst						0.145
rs424975389	*TLR5*	PIC	0.000	0.000	0.269	0.000	0.038	0.053
		H obs	0.000	0.000	0.400	0.000	0.040	0.055
		H exp	0.000	0.000	0.337	0.000	0.040	0.053
		n	20.000	20.000	20.000	60.000	100.000	220.000
		Fis	0.000	0.000	-0.250	0.000	-0.020	-0.225
		Fst						0.462
rs55631273	*TLR2*	PIC	0.373	0.269	0.375	0.374	0.277	0.361
		H obs	0.500	0.400	0.200	0.600	0.420	0.455
		H exp	0.521	0.337	0.526	0.506	0.335	0.474
		n	20.000	20.000	20.000	60.000	100.000	220.000
		Fis	-0.010	-0.250	0.600	-0.205	-0.266	0.012
		Fst						0.141
rs430457884	*TLR2*	PIC	0.223	0.373	0.375	0.000	0.038	0.177
		H obs	0.300	0.900	0.200	0.000	0.040	0.147
		H exp	0.268	0.521	0.526	0.000	0.040	0.197
		n	20.000	20.000	20.000	58.000	100.000	218.000
		Fis	-0.177	-0.818	0.600	0.000	-0.020	-0.117
		Fst						0.258
rs160821602	*TLR2*	PIC	0.223	0.000	0.000	0.000	0.038	0.044
		H obs	0.300	0.000	0.000	0.000	0.400	0.046
		H exp	0.268	0.000	0.000	0.000	0.040	0.045
		n	20.000	20.000	20.000	60.000	100.000	220.000
		Fis	-0.177	0.000	0.000	0.000	-0.020	-0.156
		Fst						0.104
rs412232316	*TLR5*	PIC	0.365	0.091	0.332	0.000	0.038	0.182
		H obs	0.200	0.100	0.600	0.000	0.040	0.100
		H exp	0.505	0.100	0.442	0.000	0.040	0.202
		n	20.000	20.000	20.000	60.000	100.000	220.000
		Fis	0.583	-0.053	-0.429	0.000	-0.020	0.091
		Fst						0.434
rs416833129	*TLR5*	PIC	0.365	0.091	0.375	0.000	0.000	0.146
		H obs	0.200	0.100	0.600	0.000	0.000	0.083
		H exp	0.505	0.100	0.526	0.000	0.000	0.160
		n	20.000	20.000	20.000	60.000	98.000	218.000
		Fis	0.583	-0.526	-0.200	0.000	0.000	0.163
		Fst						0.302

The highest observed heterozygosity, which indicated a high within-population diversity, was obtained for rs403288183-*TLR8*, rs412232316-*TLR5*, and rs416833129-*TLR5* in the Dorper population, with a value of 0.700, 0.600, and 0.600, respectively. With a similar pattern for rs424975389-*TLR5* and rs55631273-*TLR2* in the Ile de France population, with the value of 0.600 for both SNPs. The Fst value ranged from 0.104 to 0.462, indicating a moderate relationship among the observed breeds.

The HWE test (*x*^2^) is also shown in Table 3. Some SNPs have deviated from the HWE, such as the SNP rs416833129-*TLR5* in Hungarian Tsigai, rs430457884-*TLR2* in White Dorper, rs55631273 and rs430457884 of *TLR2* in Dorper, and rs55631273-*TLR2* in the Hungarian Merino population. The South African Dorper population was found to be a population with most SNPs deviating from the HWE. It was found that the Hungarian Merino population had the highest proportion of polymorphic markers (100%), and Ile de France had the lowest one (33.33%), with a fixed allele in 6 SNPs, except for rs403288183-*TLR8*, rs401390846-*TLR2*, and rs55631273-*TLR2.*

### Principal component analysis

**Fig. 1:**
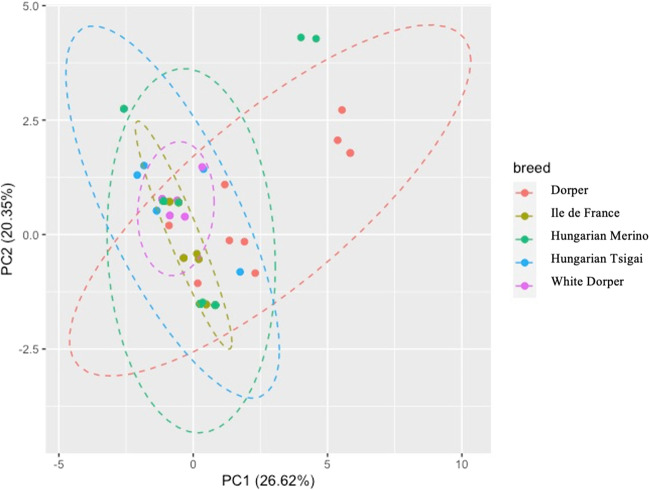
Score biplot of principal component analysis (PCA) of 9 SNPs of 110 animals from 5 different sheep breeds. Different breeds are showed in different colors

**Fig. 2:**
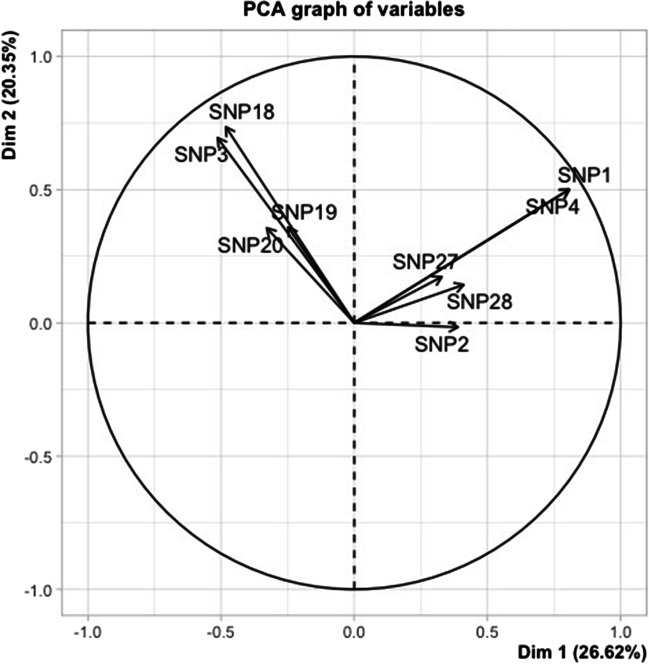
Loadings biplot of principal component analysis (PCA) of 9 SNPs of 110 sheep. SNP1 = rs429546187-*TLR5*; SNP2 = rs403288183-*TLR8*; SNP3 = rs401390846-*TLR2*; SNP4 = rs424975389-*TLR5*; SNP18 = rs55631273-*TLR2*; SNP19 = rs430457884-*TLR2*; SNP20 = rs160821602-*TLR2*; SNP27 = rs412232316-*TLR5*; and SNP28 = rs416833129-*TLR5*

The result of the PCA is shown in Figs. 1 and 2. As displayed in Fig. 1, PC1 and PC2 account for 26.62% and 20.35% of the total variation in the five breeds, respectively, with a cumulative variance of 46.97%. The PCA was unsuccessful in separating breeds based on the genetic data. Based on the PCA loadings value (Fig. 2), the rs429546187-*TLR5* (SNP1) and rs424975389-*TLR5* (SNP4) had maximum values in PC1, as they showed maximum variance.

## Discussion

This study aimed to examine the genetic polymorphism of 48 SNPs in five European and African sheep breeds reared in Hungary, with an emphasis on gastrointestinal parasite resistance, using the KASP-PCR technique. As many as 16 (33.33%) of 48 SNPs were failed for genotyping, resulting in the success rate of the KASP technique in this study (66.67%) being lower than in previous studies on similar genes in goats (Ilie et al. 2018) and genes involved in milk composition in goats (Kusza et al. 2018) Due to the fact that monomorphic markers were excluded, only 9 SNPs (18.75%) were included for further analysis.

In this study, 9 polymorphic markers located in 3 genes associated with the parasite resistance were observed, namely the *TLR5* (4 SNPs; rs429546187, rs424975389, rs412232316, and rs416833129), *TLR8* (1 SNPs; rs403288183), and *TLR2* (4 SNPs; rs401390846, rs430457884, rs55631273 and rs160821602).

When studying disease resistance in livestock, toll-like receptors (TLRs) are frequently investigated, considering that they are proteins pattern-recognition receptors (PRRs) that initiate the inflammatory processes (Ruiz-Larrañaga et al. 2011; Nie et al. 2018) and induce innate immune responses by identifying pathogen-associated molecular patterns (PAMPs) produced by pathogens such as bacteria, viruses, fungi, and parasites (Ma et al. 2011; Vijay et al. 2018). Mammalian TLRs are a large family with at least 13 members (Roach et al. 2005); however, only 10 members have been identified in sheep, even though all *TLR* genes in the species are highly comparable to caprine *TLR*, also showing over 95% similarity to bovine orthologs (Jungi et al. 2011). TLRs are present in a variety of cellular locations; for instance, *TLR2* and *TLR5* are found on the cell surface, which acts as bacterial and fungal sensors, whereas *TLR8* acts as a sensor for intercellular pathogens (e.g., viruses), being found on the membranes of intracellular vesicles such as endosomes (Takeda 2004; Kawai and Akira 2006; Schumann and Tapping 2007).

Numerous studies have implicated *TLR* genes in natural genetic resistance to a variety of diseases in sheep, including ovine paratuberculosis (Yaman 2020), *Mycoplasma pneumonia* infections (Du et al. 2020), brucellosis (Li et al. 2021), and *Haemonchus contortus* infections (Toscano et al. 2019), outlining the importance of these genes in sheep health and immune system functioning. Several polymorphisms of the *TLR* genes have also been investigated and proven to be associated with sheep health and immunity (Taylor et al. 2008; Mikula et al. 2010; Olech et al. 2021). In this study, the PIC value of rs430457884-*TLR2*, rs55631273-*TLR2*, and rs416833129-*TLR5* indicated these SNPs to some degree, informative in detecting the polymorphism among individuals of the five populations investigated. Aside from that, the loading value of PCA showed that rs429546187-*TLR5* and rs424975389-*TLR5* have a substantial influence on clustering for the Hungarian populations (Tsigai and Merino).

The different genotype and allele frequency of *TLRs* genes in this study demonstrated genetic diversity amongst Hungarian sheep breeds. These results are encouraging for future development of selection and genetic improvement programs for disease and environmental adaptation, as well as utilization for conservation efforts of Hungarian local sheep breeds. The Dorper and Hungarian Merino showed the most distinctive genotype and allele frequency for the studied SNPs compared to the other three breeds. An indigenous breed such as the Hungarian Tsigai is assumed to maintain high levels of genetic diversity, compared to commercial breeds, as indigenous breeds are usually under less selection pressure; however, in this study, a high proportion of monomorphic loci were found in the Hungarian Tsigai (*n* = 10) as well as in the Ile de France population (*n* = 30). In some instances, monomorphic loci associated with disease resistance are observed, such as Tendinopathies in the Greek native horse breed (Giantsis et al. 2020) and *Mycobacterium bovis* infection in Hostein Friesien cattle (Richardson et al. 2016). This is likely because native breeds are highly tolerant of and adaptable to a wide range of environmental circumstances has led to the evolution of disease-resistant phenotype. Although the moderate Fst value and the PCA indicated a moderate relationship among the observed breeds, no noteworthy clustering was formed from the PCA score value biplot (Fig. 1), as it was also in line with the previous finding by Loukovitis et al. (2022). This failed grouping in PCA was also affected by the small sample size and the large variation in sample size across populations.

The Hungarian Merino observed in this study showed the highest polymorphism levels among all. The breed is a commercial breed and has been developed through crossbreeding with a variety of Spanish Merino and Merino-derived breeds over years of history, which has resulted in extensive genetic admixture, results in line with those reported previously by Ciani et al. (2015), which stated that the Hungarian Merino sheep dates back over 250 years and evolved from the original fine-wool Spanish Merino rams and local semi-fine wool populations. Over the years, numerous different Merino and indigenous Hungarian breeds have contributed to the breed’s evolution. Moreover, the Hungarian Merino has been through a dynamic population expansion for the past years. To support this, a study by Loukovitis et al. (2022) has confirmed the high levels of genetic variation within the population of the Hungarian Merino.

The South African Dorper is also a widely spread commercial breed, being a hardy composite breed established by crossing the Black-headed Persian and the Dorset Horn. Due to its outstanding characteristics, such as the thermotolerance ability (Joy et al. 2020), the Dorper has gained popularity in certain European regions, including Hungary (Gavojdian et al. 2013). Since its introduction in 2007, the Dorper has been one of the most prolific breeds found in Hungary (Budai et al. 2013), although the number of purebreds in Hungary is still limited, and the ram is mainly used as terminal sires for crossbreeding with indigenous maternal breeds to increase production traits (Gavojdian et al. 2015a, b). According to some authors, the drawback of this breed to European rearing conditions would be the lower resistance to diseases, as evidenced by previous findings by Guo et al. (2016) and Estrada-Reyes et al. (2019). However, according to reports by Gavojdian et al. (2015a, b), the Dorper and White Dorper breeds have similar disease resistance and health indicators as the local Hungarian Tsigai, when reared under identical conditions.

Our current findings serve as a starting point for the characterization of European and African sheep breeds based on gastrointestinal parasitism resistance genes. They can be utilized to establish precise conservation measures aimed at improving disease resistance and monitoring the genetic variability of sheep breeds, especially those reared in Central Europe. However, populations with a larger sample size would be beneficial to obtain a better figure of the genetic diversity of the European sheep breeds.

## Conclusion

This study describes polymorphisms in parasite resistance genes in European and African sheep breeds reared in Hungary. Our study revealed nine polymorphic markers in *TLR5*, *TLR8*, and *TLR2* genes associated with parasite resistance in Hungarian Tsigai, White Dorper, Dorper, and Hungarian Merino sheep using the KASP assay, giving room for genetics selection for parasite resistance traits in Hungarian sheep population. In general, the study reported a moderate level of genetic variability and that a low to moderate within-breed diversity was maintained in the studied populations. Although these SNPs require additional research into marker associations and their marker-quantitative trait locus phase relationships in each population to precisely define each SNP effect, and the number of samples is limited in this pilot study, the results obtained may prove valuable and contribute to the future molecular marker studies on disease resistance in sheep.

## Data Availability

All data generated or analyzed during this study are included in this article.
